# Simulating the impact of preventive strategies for older persons’ emergency care demands on health care utilisation, Amsterdam as Dutch use case

**DOI:** 10.1093/ageing/afag162

**Published:** 2026-06-08

**Authors:** Oscar S Smeekes, Tim R de Boer, Hanna C Willems, Rob D van der Mei, Bianca M Buurman

**Affiliations:** Amsterdam UMC location University of Amsterdam, Internal Medicine, section of Geriatric Medicine, Meibergdreef 9, Amsterdam, Netherlands; Centrum Wiskunde & Informatica-Stochastics, Science Park 123, Amsterdam, Netherlands; Amsterdam UMC location University of Amsterdam, Internal Medicine, section of Geriatric Medicine, Meibergdreef 9, Amsterdam, Netherlands; Centrum Wiskunde & Informatica-Stochastics, Science Park 123, Amsterdam, Netherlands; Amsterdam UMC location University of Amsterdam, Internal Medicine, section of Geriatric Medicine, Meibergdreef 9, Amsterdam, Netherlands; Amsterdam UMC location Vrije Universiteit Amsterdam, Medicine for Older People, Amsterdam Public Health Research Institute, De Boelelaan 117, Amsterdam, Netherlands

**Keywords:** emergency department visits, system dynamics modelling, preventive strategies, acute, intermediate, and chronic care, older adult

## Abstract

**Background:**

Preventive strategies aim to reduce older adults’ emergency department (ED) visits and related adverse health outcomes, but their impact on healthcare system utilisation remains unclear. This can be investigated using system dynamics modelling, which uses data to explore effects and test strategies across populations.

**Aim:**

In this study, we simulated and studied how preventive measures affect acute hospitalisations, intermediate care, home care, and nursing home admissions among community-dwelling older adults after an ED visit.

**Methods:**

We developed a system dynamics simulation model tracing older adults from ED visit to hospitalisation, intermediate care, discharge home (with or without care), nursing home admission, or death within 30 days. Simulated strategies included proactive care, geriatric emergency medicine, and hospital-at-home. Data from Amsterdam residents aged ≥65 who visited the ED in 2019 were used. Patients were categorised by home care status.

**Results:**

31,049 patient journeys were used in the modelling. Of the simulated strategies, hospital-at-home demonstrated the largest potential reductions in institutionalised care use after ED visits in the total cohort: acute hospitalisations (−10.2%), intermediate care (−16.7%), nursing home care (−10.7%). Furthermore, it showed a reduction in personal home care (−1.8%), and limited increases in household help (+2%) and nursing home care at home (+1.5%).

**Conclusion:**

Of the simulated strategies, hospital-at-home reduced healthcare use post-ED most effectively, causing the greatest decrease in institutional care without requiring a meaningful increase in home care services during one year of follow-up.These findings can guide policymakers, insurers, and institutions in choosing effective preventive strategies for regional populations.

## Key Points

- Hospital-at-home was the most effective strategy in reducing overall healthcare utilisation.
- It decreased institutional care needs without requiring major increases in home care services over one year.
- These findings help guide effective preventive care strategy decisions

## Introduction

Society is ageing, and older adults with comorbidities are visiting emergency departments (EDs) more often. This has made effective emergency care an international concern [1, 2]. The use and organisation of emergency care differ substantially across countries and regions; for example, approximately 430/1000 individuals visit the ED annually in the United States, compared with 350/1000 in England, and 120/1000 in the Netherlands [1, 2]. Acute care is mainly provided by general practitioners (GPs) in the Netherlands, and GPs make 40% of ED referrals compared with 5% in England [1, 2]. In Amsterdam, the ~900,000 inhabitants are allocated across four hospitals in six locations, with specific triage criteria, whereas in Enschede, the ~160,000 inhabitants rely on a single hospital. This variation complicates the transferability of preventive strategy results across countries and regions.

Multiple preventive strategies—such as proactive care in general practise, hospital-at-home, and geriatric emergency medicine—have been developed to reduce acute hospitalisations, nursing home admissions, and death [3–10]. However, the effectiveness of these strategies across different healthcare systems and regions remains unclear [10–15]. Testing the effectiveness of these strategies in clinical practise through randomised clinical trials is not optimal because these studies are resource-intensive and without guaranteed benefit. One alternative is simulation models, which use real-world data to explore the effects of test strategies on the healthcare systems. This approach can help identify the most effective preventive strategies before they are implemented.

Simulation models have been used in multiple studies to investigate emergency care demand [16], and most of these studies have used discrete event simulation to study patient flow in the ED [16, 17]. Brailsford *et al*. [18] used system dynamics models to examine emergency and on-demand healthcare in Nottingham, England, and found that these models can capture complex systems, can estimate how strategies affect patient counts, length of stay, and bed occupancy, and can provide stakeholders with insights into preventive strategies. More recently, England *et al*. [7] showed that system dynamics models can be decision-support tools in assessing the impact of preventive strategies on outcomes after an ED visit, such as acute hospitalisation, discharge home, nursing home admission, or death.

However, these simulation models do not show how preventive strategies affect the use of intermediate and home care after an ED visit. This information is essential because these post-ED care forms not only affect burden on the healthcare system but also reflect what support older adults need after visiting the ED. Identifying those populations at a higher risk of ED visits and avoiding the crises in these populations that result in ED visits is crucial. For example, older individuals who receive home care have a higher risk of ED visits, hospitalisations, nursing home admissions, and death than older individuals who live independently do [19–22]; therefore, simulations should examine the impact of preventive strategies in individuals receiving home care.

The aim of this study is to simulate and evaluate the impact of preventive measures on acute hospitalisations, intermediate care, home care, and nursing home admissions among community-dwelling older adults living in Amsterdam after an ED visit. We chose Amsterdam because it has a diverse population, fragmented provider network, and limited insight into patient flows, and struggles to find fitting and timely emergency care for older adults [23].

## Methods

### Design and population

For this simulation study, we developed a system dynamics model that follows older adults from their ED visit to 30 days after discharge. We collected data on the demographics (age, gender, living situation, income, and socioeconomic status score), deaths, and healthcare claims of individuals living in Amsterdam. These data were obtained from the Statistics Netherlands database, which anonymously compiles data that is routinely collected from the government, municipalities, and health insurers for policy evaluation and research purposes [24]. We used these claims data because they provide a representative view of longitudinal care trajectories across settings.

The need for ethical approval was waived according to the guidelines of the Dutch Central Commission for Human Subjects Research [25] and the Declaration of Helsinki [26]. Registries and codes are reported in Appendices 1–2; details of the Amsterdam area, home care, and the Dutch healthcare system are described in Appendix 3; and background on system dynamics models is included in Appendix 4. Patients were included if they were ≥ 65 years of age, community dwelling, and had visited the ED. We studied older adults presenting to the ED, as they represent a clearly identifiable high-risk population for subsequent healthcare use and adverse health outcomes [27, 28].

### The model

Our simulation model is illustrated in Figure 1a and shows the flow of our participants through the emergency system, from the ED visit to possible hospitalisation, intermediate care admission, discharge, return home with or without home care, nursing home admission, or death 7–30 days after discharge. We categorised our patients into groups based on the home care they received in the 30 days before the ED visit. These care groups were no home care, household help, personal care with or without household help, or nursing home care at home. In each category, we assessed how three preventive strategies affected outcomes after the ED visit.

**Figure 1:**
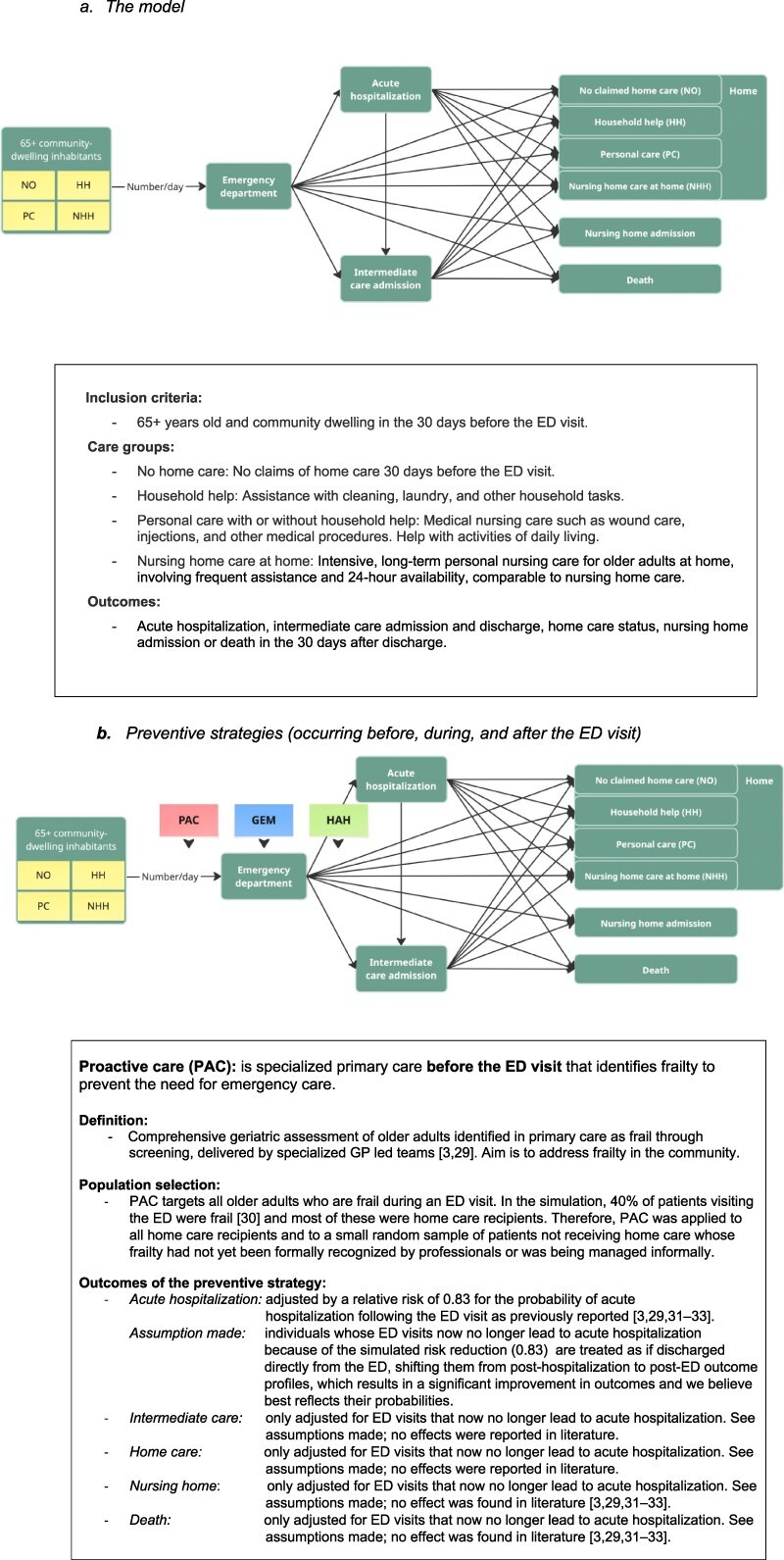
The simulation model and tested preventive strategies. (a) The model, (b) preventive strategies (occurring before, during, and after the ED visit). Abbreviations: ED = emergency department; GEM = geriatric emergency medicine; GP = general practitioner; HAH = hospital-at-home; PAC = proactive care. Boxes represent the average number of visits made per day in a certain location. Arrows represent average fractions of these numbers that are transimited to the next location.

**Figure 1:**
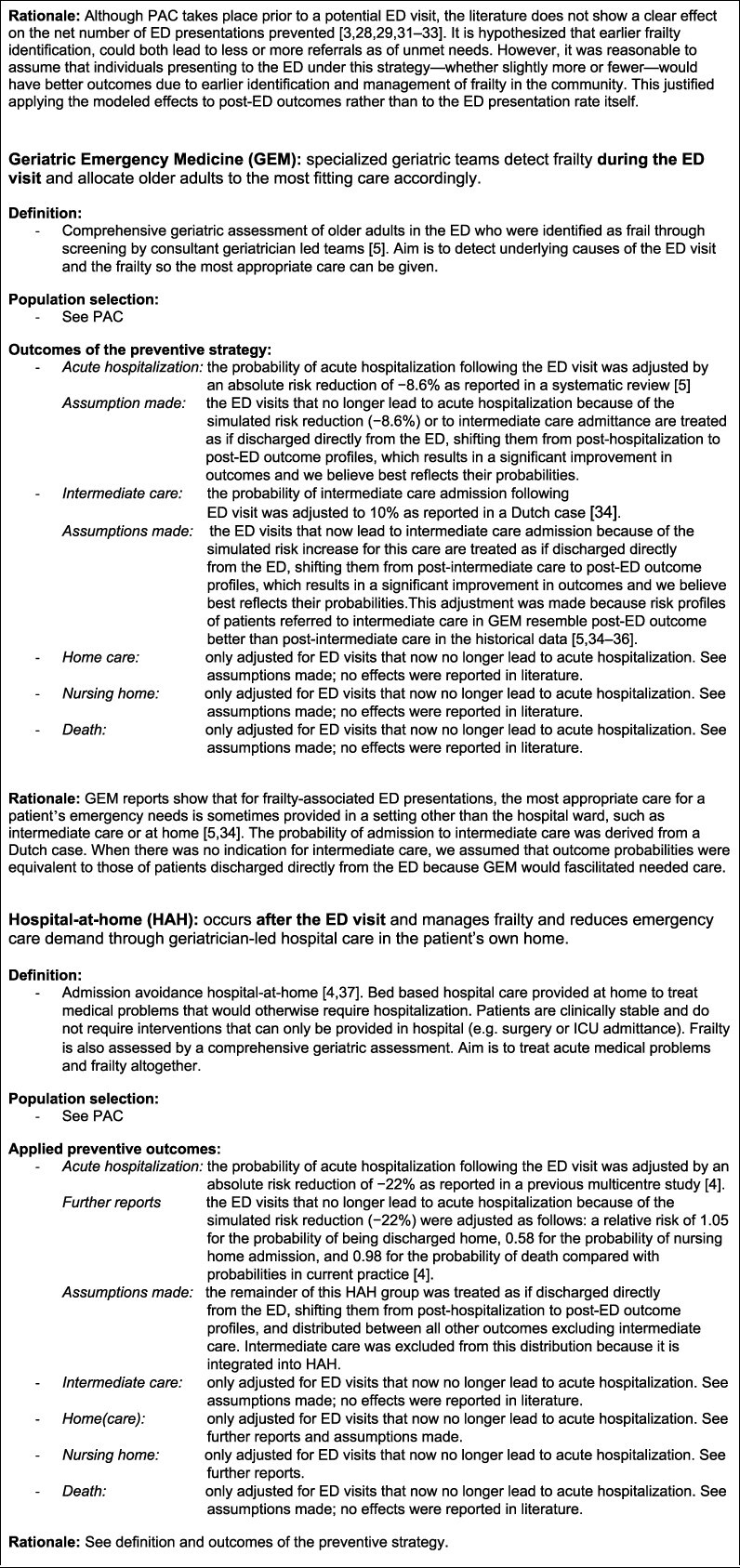
Continued.

### The preventive strategies

We selected three preventive strategies—proactive care (PAC) before the ED visit, geriatric emergency medicine (GEM) during the ED visit, and hospital-at-home (HAH) after the ED visit—because they are established approaches in geriatric care and have been modelled in a prior simulation study [7]. These strategies aim to prevent acute hospital admissions and related adverse health outcomes, such as functional decline, delirium, and complications, through comprehensive geriatric assessment, thereby reducing healtcare utilisation [4, 5, 28, 29]. However, they differ in timing, setting, and the care teams involved. Comparing strategies at these different points in the patient journey allowed us to explore how timing and context may influence effectiveness and feasibility in a local healthcare system, providing insights into which approach might best reduce health care utilisation among older adults visiting the ED. As these strategies are interpreted and implemented differently across international settings, we provide clear definitions, outlined in the textboxes in Figure 1b.

These preventive strategies were operationalised for simulation in the model through a stepwise process. First, a literature search was conducted to identify studies reporting outcomes included in our model. Given that a prior simulation study had already systematically identified evidence, this served as the primary basis for our analysis [7]. Only studies reporting outcomes that could be directly incorporated into our model were considered, reflecting a model-driven selection. Second, a structured group discussion with the Data-driven Optimisation for a Vital Elderly Care System (DOLCE VITA) Research Group, consisting of geriatricians, data scientists, and modelling experts, reviewed all parameters and outcomes. The group discussed whether literature-reported outcomes were applicable locally. For outcomes that were missing or not directly applicable, the group discussed whether a reasonable assumption could be made. If so, outcomes were adjusted accordingly; if not, they were labelled as ‘not adjusted’ or ‘only adjusted for …’ to indicate indirect effects from adjustment of other outcomes. All reported outcomes and assumptions are documented in the textboxes in Figure 1b.

### Demographics

We collected data on age, gender, living situation, income, and socioeconomic status of our participants when they visited the ED in 2019. We used the Statistics Netherlands definitions for income class and socioeconomic status [38]. Income class was defined as a household’s income as a percentage of the social minimum. Socioeconomic status was assessed using the composite SES-WOA score, which is based on income, education, and employment, and ranges from −2 to 1, with 0 being the national average for Dutch adults [39]. We also collected ATC-4 codes, which indicate the number of prescribed drugs per individual.

### Statistical analysis

Chi-square tests were used to compare baseline dichotomous variables between groups. Initially, *t*-tests were used to compare continuous variables from the no home care group with those from each home care group separately. Then, one-way ANOVA was used to compare the different home care groups.

### Model validation and simulation

The model was internally validated by comparing simulation results with summary statistics derived from the model’s input parameters that were taken from the Statistics Netherlands database (see Appendix 5 for further details on the validation process). The model used the average number of older adults visiting the ED per group per day as input for each simulation run. The probabilities of transitioning to a next station, for example from ED to acute hospitalisation and acute hospitalisation to nursing home, were pre-calculated based on the input data. The probability of these transitions changes when a preventive strategy is run; for example, fewer older adults will be acutely hospitalised if the PAC strategy is run and those adults that are no longer hospitalised will be reassigned to other outcomes as described in Figure 1b. These redirections in patient flow lead to new average numbers of older adults per station per day, and in turn per year.

## Results

### Population characteristics

At baseline, community-dwelling individuals aged 65 and older made 31 049 visits to the ED in Amsterdam (Table 1a). Of these visits, 20 000 (64.4%) were by patients from the no home care group, 3262 (10.5%) were from the household help group, 6934 (22.3%) were from the personal care group, and 853 (2.7%) were from the nursing home care at home group. Adults in the no home care group were generally younger than those in the home care groups and more likely to be male than female and live alone. Adults in this group also had a higher SES-WOA score, earned a higher income, and used less medication.

**Table 1:** (a) Population characteristics per group for community-dwelling older adults visiting the ED in Amsterdam in 2019; (b) annual use of care by community-dwelling older adults after visiting the ED in Amsterdam in 2019 (before simulation).

a.						
Population characteristics	Total cohort	NO	HH	PC	NHH	*P*-values
ED visits	31,049 (100%)	20,000 (64.4%)	3262 (10.5%)	6934 (22.3%)	853 (2.7%)	< .001
Age (mean in years) [SD]	77.1 [7.9]	75.2 [7.0]	78.9 [7.7]	80.8 [8.3]	84.8 [8.3]	< .001
Female gender (f%)	52.1%	46.6%	72.5%	56.9%	62.0%	^*^< .001
Lives alone (%)^a^	54.1%	45.3%	80.7%	65.2%	68.4%	^*^< .001
Socioeconomic status score^b^ (mean) [SD]	−0.5 [0.7]	−0.3 [0.7]	−0.8 [0.5]	−0.6 [0.6]	−0.6 [0.6]	< .001
Income^c^ (mean) [SD]	181.0 [131.8]	204.1 [145.2]	125.0 [60.0]	145.2 [95.6]	144.4 [118.4]	< .001
Medication^d^ (mean) [SD]	10.6 [5.7]	9.4 [5.3]	12.0 [5.4]	13.3 [5.7]	11.0 [5.6]	< .001
b.						
Annual care use after ED visits (before simulation)	Total cohort (65+ at home)	NO	HH	PC	NHH	*P*-values
Acute hospitalisation	13,911 (44.8%)	7989 (39.9%)	1511 (46.3%)	3922 (56.5%)	489 (57.3%)	< .001
Intermediate care admission	2125 (6.8%)	992 (5.0%)	287 (19.0%)	803 (11.6%)	43 (5.0%)	< .001
No claimed home care	17,424 (56.1%)	16,534 (82.7%)	153 (4.7%)	710 (10.2%)	27 (3.2%)	< .001
Household help	2493 (8.0%)	109 (0.5%)	2105 (64.5%)	252 (3.6%)	27 (3.2%)	< .001
Personal care	7485 (24.1%)	2043 (10.2%)	694 (21.3%)	4693 (67.7%)	55 (6.4%)	< .001
Nursing home care at home	626 (2.0%)	61 (0.3%)	37 (1.1%)	121 (1.7%)	407 (47.7%)	< .001
Nursing home admission	765 (2.5%)	226 (1.1%)	57 (1.7%)	265 (3.8%)	217 (25.4%)	< .001
Death	2256 (7.3%)	1027 (5.1%)	216 (6.6%)	894 (12.9%)	119 (14.0%)	< .001

Abbreviations: NO = no home care, HH = household help, PC = personal care with or without household help, NHH = nursing home care at home. ^a^The socioeconomic status score is a mean composite score of income, education, and employment and ranges from − 2 to 1, with a lower score corresponding to a lower income, education, and/or employment. ^b^The mean socioeconomic status score in Dutch adults is 0 [39]. ^c^Income is reported as a percentage of the social minimum, where 100 corresponds to a mean income equal to the social minimum. ^d^Medication is the mean cumulative use of distinct prescribed drugs over a year based on a 2019 ATC-4 code. *P*-values with a ^*^ were calculated using the Chi-square test, *P*-values without ^*^ were calculated using ANOVA tests. The variables tested with ANOVA were tested between the home care groups and the NO group, and all *P*-values were < .001.

### Health care use after the emergency department

**Figure 2:**
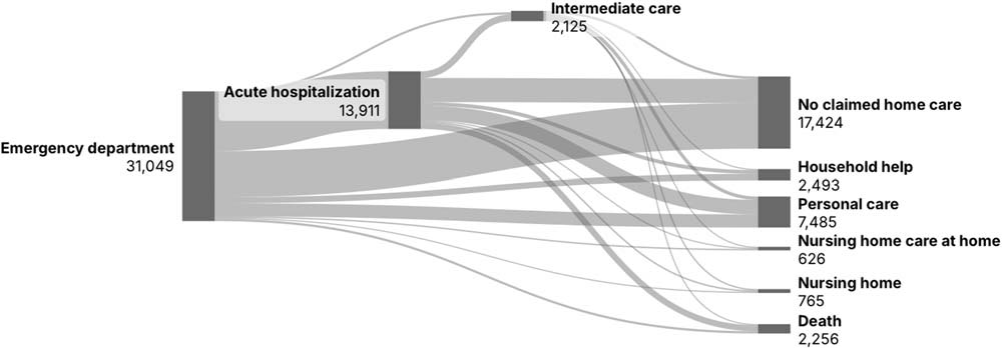
Patient flow from the emergency department to post-emergency care before simulation.

We calculated post-ED care use rates by care group before simulation (Table 1b). Acute hospitalisation, intermediate care, and nursing home admission rates were lowest in the no home care group (39.9%) and higher in all home care groups. Acute hospitalisation was highest in the personal care (56.5%) and nursing home care at home groups (57.3%); intermediate care in the household help group (19.0%); and nursing home admission in the nursing home care at home group (47.7%). Transitions to more intensive care were most common in the household help group (21.3% to personal care), while transitions to less or no care were highest in the personal care group (10.2%), suggesting reversibility. Despite lower rates, the no home care group contributed the most acute hospitalisations (*n* = 7989) and intermediate care admissions (*n* = 992) due to its size; nursing home admissions were highest from the personal care group (*n* = 265). Care trajectories are shown in Figure 2.

### Health care use before and after simulation in the total cohort

We investigated the simulated impact of PAC, GEM, and HAH on post-ED healthcare use in the total cohort (Table 2a). HAH showed the largest reductions in acute hospitalisation (−10.2% vs −8.3% PAC, −4.0% GEM), intermediate care (−9.2% vs −4.3% PAC, +40% GEM), personal care (−0.4% vs +0.8% PAC, +0.5% GEM), and nursing home admissions (−7.0% vs −5.0% PAC, −3.0% GEM). However, HAH also increased household help (2.4% vs 2.3% PAC, 1.3% GEM) and nursing home care at home (2.3% vs 3.1% PAC, 1.5% GEM). PAC yielded the greatest mortality reduction (−7.0% vs −2.7% HAH, −4.1% GEM).

**Table 2:** Care use before and after simulation for the total cohort (a) and for older adults receiving personal home care (b).

a. Total cohort							
Health care use per year	Before	After PAC	% change^a^	After HAH	% change^a^	After GEM	% change^a^
Acute hospitalisations	13,911 (44.8%)	12,762 (41.1%)	−8.3%	12,488 (40.2%)	−10.2%	13,355 (43.0%)	−4.0%
Intermediate care admissions	2125 (6.8%)	2033 (6.5%)	−4.3%	1930 (6.2%)	−9.2%	2976 (9.6%)	+40.0%
No home care	17,424 (56.1%)	17,429 (56.1%)	0.0%	17,402 (56.0%)	−0.1%	17,421 (56.1%)	0.0%
Household help	2493 (8.0%)	2549 (8.2%)	+2.3%	2553 (8.2%)	+2,4%	2524 (8.1%)	+1.3%
Personal care	7485 (24.1%)	7545 (24.3%)	+0.8%	7517 (24.2%)	−0.4%	7522 (24.2%)	+0.5%
Nursing home care at home	626 (2.0%)	646 (2.1%)	+3.1%	641 (2.1%)	+2.3%	636 (2.0%)	+1.5%
Nursing home admissions	765 (2.5%)	727 (2.3%)	−5.0%	711 (2.3%)	−7.0%	742 (2.4%)	−3.0%
Death	2256 (7.3%)	2153 (6.9%)	−4.6%	2224 (7.2%)	−1.4%	2203 (7.1%)	−2.4%
b. Personal care group							
Health care use per year	Before	After PAC	% change^a^	After HAH	% change^a^	After GEM	% change^a^
Acute hospitalisations	3922 (56.5%)	3255 (46.9%)	−17.0%	3059 (44.1%)	−22.0%	3585 (51.7%)	−8.6%
Intermediate care admissions	803 (11.6%)	750 (10.8%)	−6.6%	673 (9.7%)	−16.2%	1233 (17.8%)	+53.5%
No home care	710 (10.2%)	697 (10.1%)	−1.8%	685 (9.9%)	−3.6%	703 (10.1%)	−1.0%
Household help	252 (3.6%)	243 (3.5%)	−3.4%	238 (3.4%)	−5.4%	247 (3.6%)	−1.8%
Personal care	4693 (67.7%)	4802 (69.3%)	+2.3%	4791 (69.1%)	+2.1%	4754 (68.6%)	+1.3%
Nursing home care at home	121 (1.7%)	117 (1.7%)	−3.0%	115 (1.7%)	−4.9%	119 (1.7%)	−1.6%
Nursing home admissions	265 (3.8%)	248 (3.6%)	−6.2%	235 (3.4%)	−11.0%	253 (3.6%)	−4.4%
Death	894 (12.9%)	827 (11.9%)	−7.5%	870 (12.5%)	−2.7%	858 (12.4%)	−4.1%

^a^% change is the percentage of cases where the number of outcomes changes after the preventive strategy. Therefore, when numbers are small, percentages can be high although the strategy has limited impact and vice versa. Numbers are rounded to the nearest whole visit. Abbreviations: ED = emergency department; GEM = geriatric emergency medicine, HAH = hospital-at-home, PAC = proactive care.

### Health care use before and after simulation in the personal care group

We evaluated the simulated impact of PAC, GEM, and HAH on post-ED healthcare use in the personal care group (Table 2b), because this group was both large and at higher risk for al outcomes than the other groups (Table 1). HAH produced the greatest reductions in acute hospitalisation (−22.0% vs −17.0% PAC, −8.6% GEM), intermediate care (−16.2% vs −6.6% PAC, +53.7% GEM), household help (−5.4% vs −3.4% PAC, −1.8% GEM), nursing home care at home (−4.9% vs −3.0% PAC, −1.6% GEM), and nursing home admissions (−11.0% vs −6.2% PAC, −4.4% GEM). However, HAH increased personal care use (+2.1% vs +2.3% PAC, +1.3% GEM). PAC again showed the largest mortality reduction (−7.5% vs −2.7% HAH, −4.1% GEM).

## Discussion

In this simulation study, we studied the impact of three preventive strategies on the use of acute, intermediate, and chronic care by older individuals after an ED visit in Amsterdam using historical data. All simulated strategies reduced care use after ED visits particularly among older adults who received nurse-based personal home care before their ED visit.

### Impact of the preventive strategies

Of the simulated strategies, HAH caused the largest reduction in use of acute, intermediate, and chronic care without meaningfully increasing use of household help, personal care, and nursing home care at home during one year of follow-up. We have two explanations for this effect. The first explanation is that HAH reduced the number of acute hospitalisations, which in turn reduced the need for further intermediate care, nursing home care, and home care that are typically associated with an acute hospitalisation. The second explanation is that HAH integrates home, intermediate, and hospital care into a new service, which reduces the need for these separate services, whereas PAC and GEM redirect patients from the hospital to these services. PAC involves primary care before the ED visit, so is not visible in the model, which begins at ED presentation. However, GEM is reflected in the model because it redirects patients who do not need to be hospitalised to rehabilitation services during their ED visit, which increases the use of intermediate care.

All three strategies marginally increased the use of home care, which is understandable because older adults who otherwise would have died or been admitted to a nursing home then returned home after their emergency care. However, this slight increase in home care was modest compared with the more striking reductions in institutionalised care.

In the literature, how HAH, PAC, and GEM affect intermediate and home care has not been well described. HAH has been shown to increase ownership and understanding of illness in the home setting, making older adults more self-sustaining and providing an effective alternative to intermediate care [4, 40]. Another advantage of HAH is that older adults are more physically active at home than they are during institutionalisation [41–43]. PAC identifies frail individuals before an ED visit, which has been shown to improve outcomes in older adults, including less functional decline, improved quality of life, and better health management [14, 28, 31]. However, this early identification of frailty can increase the demand for primary care because it exposes needs that were previously unmet, leading to more consultations, medication reviews, and referrals [28]. In a Dutch hospital implementing GEM, previously unrecognised frailty was uncoverd in 20% of consultations, which had a significant impact on the individuals [34]. These individuals are usually referred to intermediate care or home care rather than the hospital, and advanced care planning is initiated to avoid unnecessary emergency care visits in the future [34]. The results of our simulations were in line with these earlier findings, demonstrating a broader effect in a different cohort.

### Finding the optimal strategy

Taken together, our findings and those in the literature [19, 42, 43] suggest that preventing acute hospitalisation is a key mechanism for preventing the need for hospital and nursing home care after an ED visit. This is because acute hospitalisation primarily addresses somatic issues and often overlooks nutrition, mobility, and social needs, which need to be met to avoid functional decline, complications, and increased care dependence after discharge [19, 42, 43]. Avoiding acute hospitalisations can be achieved in clinical practise by approaches such as HAH, PAC and GEM. However, choosing the right strategy for the right setting remains a key challenge. Our model suggests that a promising approach is to apply these interventions to specific high-risk populations, such as those receiving (nurse-based) personal home care. This group constitutes a substantial subpopulation (22.3%) and is relatively easy to identify by the professional home care they receive. In this population, we found that HAH reduced institutional care twofold without increasing the need for other forms of home care. This large reduction reflects the high baseline risk of this population requiring such services after an ED visit, and thereby the greater potential for prevention. With daily professional care and partly reversible frailty, these individuals can benefit greatly from preventive interventions [21, 22].

Another determinant in deciding which preventive interventions are feasible and effective is the local care infrastructure. For example, for HAH to function effectively, sufficient home care services, digital monitoring, and established communication with the local hospital are needed. This is the case in the Dutch city of Alkmaar**,** where HAH is used to manage patients with heart failure and COPD [44]. For GEM to function effectively, a strong geriatric team is needed to support the local ED with primary care and intermediate care services [34]. These tools can reshape care pathways to improve patient outcomes and maintain system sustainability. Our findings underscore the need for an integrated, chain-wide perspective when relocating patient care based on their needs.

### Limitations

This study has several limitations that should be considered. First, our findings are specific to older adults presenting to the ED and may not fully generalise to community-dwelling older adults who seek acute care in other settings, such as primary care. Second, we tried to estimate the probabilities of outcomes that could not be directly measured—such as intermediate care use and home care demand—based on the best available assumptions. However, the accuracy of these estimates cannot be validated because empirical data on their effects in the Dutch health care system is lacking. This highlights an important area for future research. Third, although our model offers a broader view than earlier models of how these preventive strategies may reduce healtcare use after an ED visit, it does not yet capture the feedback effects on how these changes may influence subsequent ED visits, including their volume, case mix and outcomes. For instance, early intensification of intermediate or home care for older adults may help prevent subsequent ED visits and acute hospital admissions [45, 46], which may lead to a case mix with fewer frailty-related problems upon presentation to the ED. We believe that these feedback effects could amplify the preventive impact of our modelled strategies. Fourth, there was no representative primary care data, so this could not be included in our model; this is primarily relevant to our results on the PAC strategy.

### Future research and implications

Further studies should assess the impact of these preventive strategies on the use of intermediate care, home care services and primary care services in different regions, and should also identify the factors that determine their effectiveness. This is vital for two reasons: first, because these strategies may have feedback effects, and second, because this will inform more accurate simulation models that better reflect care pathways and resource needs. With improved data, such models can support regional collaboration between hospitals, nursing homes, intermediate care providers, home care organisations, and municipalities. This would enable stakeholders to identify and implement the most appropriate preventive strategies for their specific region.

## Conclusion

Simulating the impact of preventive strategies on the use of acute, intermediate, and chronic care after an ED visit showed that these strategies can substantially reduce the need for institutional care, and marginally increase the need for home care (including household help, personal care, and intensive nursing care at home comparable to nursing home care). These effects were most pronounced in older individuals who received personal nurse-based home care before their ED visit, identifying these individuals as a high-risk population. Of the simulated strategies, HAH had the greatest impact, and caused the largest reduction in acute hospitalisations and other forms of care, without considerably increasing the need for home care. Our findings will help policymakers, insurers, and institutions to decide which preventive strategy is most effective for which populations in their region. Future research should identify the factors that influence the success of preventive strategies to improve regional healthcare in the future.

## Supplementary Material

aa-25-3311-File002_afag162
