# Supplementary material for: Simulating the impact of preventive strategies for older persons’ emergency care demands on health care utilisation, Amsterdam as Dutch use case
Source: Age Ageing. 2026 Jun 8;55(6):afag162. doi: 10.1093/ageing/afag162 (PMC13245184; doi:10.1093/ageing/afag162)
Supplement: aa-25-3311-File002_afag162 [file aa-25-3311-file002_afag162.docx]

**Appendices 1-5 registries, codes, and more information on: Amsterdam metropolitan area, home care, the Dutch healthcare system, background on system dynamics models and validation process of the model**

Simulating the impact of preventive strategies for older persons’ emergency care demands on health care utilization

*Amsterdam as Dutch use case*

1. Registries

2. Codes

3. The Amsterdam metropolitan area, home care and the Dutch healthcare system

4. Background on system dynamics models

5. More information on the validation process of the model

1. **Registries**

The following databases of CBS were used for this paper (description of data included in these registries is in Dutch):

- GBAHUISHOUDENBUS

<https://www.cbs.nl/nl-nl/onze-diensten/maatwerk-en-microdata/microdata-zelf-onderzoek-doen/microdatabestanden/gbahuishoudensbus-huishoudenskenmerken>

- MEDICIJNTAB

<https://www.cbs.nl/nl-nl/onze-diensten/maatwerk-en-microdata/microdata-zelf-onderzoek-doen/microdatabestanden/medicijntab-geneesmiddelen-op-atc-code--4-->

- INHATAB

<https://www.cbs.nl/nl-nl/onze-diensten/maatwerk-en-microdata/microdata-zelf-onderzoek-doen/microdatabestanden/inhatab-inkomen-van-huishoudens>

- KOPPELPERSOONHUISHOUDEN
- GBAOVERLIJDENTAB

<https://www.cbs.nl/nl-nl/onze-diensten/maatwerk-en-microdata/microdata-zelf-onderzoek-doen/microdatabestanden/gbaoverlijdentab-datum-van-overlijden-van-personen-ingeschreven-in-het-gba>

- SESWOA

<https://www.cbs.nl/nl-nl/onze-diensten/maatwerk-en-microdata/microdata-zelf-onderzoek-doen/microdatabestanden/seswoa-sociaaleconomische-statusscores-huishoudens>

- WLZINTAB

<https://www.cbs.nl/nl-nl/onze-diensten/maatwerk-en-microdata/microdata-zelf-onderzoek-doen/microdatabestanden/wlzzintab-personen-metgebruik-wlz-zorg-in-natura>

- GEBWMOTAB

<https://www.cbs.nl/nl-nl/onze-diensten/maatwerk-en-microdata/microdata-zelf-onderzoek-doen/microdatabestanden/gebwmotab-personen-met-wmo-maatwerkvoorzieningen>

- ZVWWVPTAB

<https://www.cbs.nl/nl-nl/onze-diensten/maatwerk-en-microdata/microdata-zelf-onderzoek-doen/microdatabestanden/zvwwvptab-personen-met-gebruik-van-zvw-wijkverpleging>

- GBAPERSOONKTAB

<https://www.cbs.nl/nl-nl/onze-diensten/maatwerk-en-microdata/microdata-zelf-onderzoek-doen/microdatabestanden/gbapersoonktab-persoonskenmerken-beperkt-in-de-brp>

- MSZZorgactiviteitenVEKTTAB

<https://www.cbs.nl/nl-nl/onze-diensten/maatwerk-en-microdata/microdata-zelf-onderzoek-doen/microdatabestanden/mszzorgactiviteitenvekttab-zorgactiviteiten-diagnose>

- ZVWELVTAB

<https://www.cbs.nl/nl-nl/onze-diensten/maatwerk-en-microdata/microdata-zelf-onderzoek-doen/microdatabestanden/zvwelvtab-gebruikmaken-van-kortdurend-eerstelijnsverblijf>

1. **Used codes**

- Postal codes of Amsterdam for selection of inhabitants
- Household help (WMO codes): 006,007,100,101,102,103,104,105,107,711
- Personal care (ZVW codes): 1, 2, 3 ,4
- Nursing home care at home (WLZ codes): 2 and 3
- Acute hospitalization codes: clinical nursing day within 24hours after 19015 (ED admittance) or 19016 (acute admittance outside ED)
- Intermediate care code: 194804 + ZVWELVTAB
- Institutionalization (WLZ codes): 1, 11 and 12.

1. **The Amsterdam metropolitan area, home care and the Dutch healthcare system**

The Amsterdam metropolitan area, home to approximately 900,000 inhabitants—including around 100,000 individuals aged 65 years or older—represents a densely populated and well-integrated healthcare region [1]. Patients in Amsterdam are distributed across four hospital organizations with six emergency department (ED) locations, following coordinated triage protocols to optimize capacity and continuity of care. The Dutch healthcare system is organized into three statutory frameworks that structure access to and funding of home and medical care: the Social Support Act (Wet maatschappelijke ondersteuning, Wmo)[2], the Health Care Insurance Act (Zorgverzekeringswet, Zvw)[3], and the Long-Term Care Act (Wet langdurige zorg, Wlz)[4]. The Wmo, administered by municipal governments and funded through social taxes, provides non-medical household support to promote independent living for those with functional limitations. The Zvw, managed by private but government-regulated health insurers, covers personal and nursing care delivered at home by district nurses, financed through mandatory health insurance premiums and social contributions. More complex and continuous care needs fall under the Wlz, which provides around-the-clock nursing home-level care at home or in institutions, coordinated by regional care offices and funded by social taxation with income-dependent co-payments. For acute medical services such as ED visits and hospital admissions, all Dutch residents are required to maintain basic health insurance, with an annual deductible of €385, beyond which care is fully covered [3].

In addition to home and long-term care, intermediate care services play an important role in the Dutch post-acute landscape. Eerstelijnsverblijf (ELV) provides short-term residential care for patients who are medically stable but temporarily unable to return home, typically following hospitalization or during recovery from acute illness. ELV is funded under the Health Care Insurance Act and aims to prevent unnecessary hospital stays or premature admissions to long-term care facilities [3]. Geriatric rehabilitation care (Geriatrische Revalidatiezorg, GRZ), also financed under the Zvw, offers multidisciplinary rehabilitation for older adults after acute hospital treatment (e.g., stroke, fracture, or severe infection), supporting functional recovery and return to independence [3]. Both ELV and GRZ are essential components of transitional and integrated care pathways within the Dutch healthcare system, bridging hospital and home care while promoting efficient resource utilization and patient-centered recovery.

1. **Background on system dynamics models**

System dynamics models provide insight into the key mechanisms of a system using simple models and simulations. This offers a user-friendly basis for stakeholder discussions and policy decisions. System dynamics models have been widely used to study the effects of interventions in business, economics, and healthcare [5,6]. In these models, key elements of the system are represented as ‘stocks’ (quantities that accumulate over time such as the number of older adults in an ED at 10 a.m.) and ‘flows’ (quantities that change over time such as the number of older adults arriving at an ED each hour) [7]. These elements are captured in differential equations to calculate how changes in one element (such as admission rates) affect the rest of the system.

1. **More information on the validation process of the model**

To ensure that the model accurately represents real-world processes, validation is essential. A valid model should reproduce the behavior of the actual system when supplied with real-world input data. Both internal and external validation can be applied. In this study, only internal validation was possible because no external datasets were available that reported the number of emergency department (ED) visits and subsequent outcomes among older adults in Amsterdam.

Internal validation involved testing whether the model could reproduce observed outcomes—such as the number of acute hospital admissions, intermediate care placements, and nursing home admissions—recorded in the Statistics Netherlands dataset. When the model was run using the average daily number of ED visits among older adults in Amsterdam in 2019, the simulated results matched the historical data precisely (see Table 1.). This exact correspondence was expected and required, as the model is structured as a flow diagram that simulates patient transitions using the 2019 data transition probabilities.

*Table 1. Internal validation of the model.*

| **Model output parameters** | **Model simulation of the total cohort** | **Internal validation, Statistics Netherlands data** |
| --- | --- | --- |
| Acute hospitalizations | 13,911 | 13,911 |
| Intermediate care admitances | 2,125 | 2,125 |
| No claimed home care use | 17,424 | 17,424 |
| Household help use | 2,493 | 2,493 |
| Personal care use | 7,485 | 7,485 |
| Nursing home care at home use | 626 | 626 |
| Nursing home admittances | 765 | 765 |
| Death | 2,256 | 2,256 |

References

1. GGD Amsterdam. Health Monitor Adults and Older Adults 2024 – Amsterdam and Amstelland. GGD Amsterdam. Available at: https://www.ggd.amsterdam.nl/onderzoek/gezondheidsmonitors/volwassenen-ouderen/#hdde73001-a9f8-4557-a6fd-78be828be7d1 (accessed 4 Sep 2025). n.d. https://www.ggd.amsterdam.nl/beleid-onderzoek/gezondheidsmonitors/amsterdamse/#hdde73001-a9f8-4557-a6fd-78be828be7d1 (10 Oct. 2023, date last accessed).

2. Dutch Government. Social Support Act (Dutch). 2015. https://wetten.overheid.nl/BWBR0035362/2024-01-01 (2 Jan. 2024, date last accessed).

3. Dutch Government. Health Care Insurance Act (Dutch). 2006. https://wetten.overheid.nl/BWBR0018450/2024-01-01 (2 Jan. 2024, date last accessed).

4. Dutch Government. Long-Term Care Act (Dutch). 2015. https://wetten.overheid.nl/BWBR0035917/2024-01-01 (2 Jan. 2024, date last accessed).

5. Darabi N, Hosseinichimeh N. System dynamics modeling in health and medicine: a systematic literature review. *Syst Dyn Rev* 2020;**36**(1):29–73. https://doi.org/10.1002/sdr.1646.

6. Haji A, Saryazdi G, Ghatari AR *et al.* Group Model Building: A Systematic Review of the Literature. *Journal of Business School* 2020;**3**(3):98–136. https://doi.org/10.26677/TR1010.2021.631.

7. Aronson D, Angelakis D. Step-By-Step Stocks and Flows: Improving the Rigor of Your Thinking. https://thesystemsthinker.com/step-by-step-stocks-and-flows-improving-the-rigor-of-your-thinking/ (last accessed 1 June 2022). n.d. https://thesystemsthinker.com/step-by-step-stocks-and-flows-improving-the-rigor-of-your-thinking/ (22 Apr. 2022, date last accessed).
